# Influence of pneumoperitoneum and head-down maneuver on the cerebral microvasculature in rabbits

**DOI:** 10.1186/s12871-022-01911-2

**Published:** 2022-12-01

**Authors:** Hiroki Kobayashi, Nobumasa Asano, Daisuke Kondo, Noriyuki Shintani, Masakazu Kotoda, Toru Matsuoka, Tadahiko Ishiyama, Takashi Matsukawa

**Affiliations:** 1 Department of Anesthesiology, Faculty of Medicine, University of Yamanashi, 1110 Shimokato, Chuo, Yamanashi, 409-3898 Japan; 2 Department of Anesthesiology, Yamanashi Prefectural Central Hospital, 1-1-1 Fujimi, Kofu, Yamanashi, 400-8506 Japan; 3 Department of Anesthesiology, Kofu Municipal Hospital, 366 Masutsubo, Kofu, Yamanashi, 400-0832 Japan; 4 Surgical Center, University of Yamanashi Hospital, University of Yamanashi, 1110 Shimokato, Chuo, Yamanashi, 409-3898 Japan

**Keywords:** Pneumoperitoneum, Cerebral microcirculation, Brain edema

## Abstract

**Background:**

With recent advances in robot-assisted techniques, an increasing number of surgeries are being performed with pneumoperitoneum and head-down maneuver (HDM) that may affect the cerebral microcirculation. For the first time, this study investigated the direct influence of pneumoperitoneum and HDM on the cerebral microvasculature in rabbits.

**Methods:**

Adult male rabbits were randomly allocated to the following groups (*n* = 7 each): control, pneumoperitoneum alone (P), and pneumoperitoneum with HDM (P + HDM) for 120 min. A closed cranial window was installed above the parietal bone to visualize the pial microvasculature. Pial arteriolar diameter and hemodynamic and blood gas parameters were measured during the 140-min observation period. Brain edema was assessed by evaluation of the brain water content at the end of the experiment.

**Results:**

Rabbits in the P and P + HDM groups exhibited a similar degree of immediate pial arteriolar dilation following the initiation of both P and P + HDM (P: 1.11 ± 0.03, *p* = 0.0044 and P + HDM: 1.07 ± 0.02, *p* = 0.0004, relative changes from the baseline value by defining the baseline as one). In the P + HDM group, pial arteriole diameter returned to the baseline level following the discontinuation of pneumoperitoneum and HDM (1.05 ± 0.03, *p* = 0.0906, vs. baseline). In contrast, the pial arterioles remained dilated as compared to the baseline level in the P group after discontinuation of pneumoperitoneum. There were no changes in pial arteriole diameter in the animals in the control group. Heart rate, blood gas parameters, and brain water content were not significantly different between the groups.

**Conclusion:**

The pial arterioles dilated immediately after pneumoperitoneum with or without HDM. The pial arterioles remained dilated 20 min after discontinuation of pneumoperitoneum alone but constricted upon discontinuation of pneumoperitoneum plus HDM. Pneumoperitoneum and HDM for 2 h did not cause brain edema.

## Background

With advances in robot-assisted techniques, an increasing number of laparoscopic surgeries are being performed. Laparoscopic surgeries have clinical advantages over conventional open surgeries, including faster postoperative recovery, fewer complications, less blood loss, reduced pain, shorter hospital stay, and better patient satisfaction [1–5].

However, in addition to insufflation of the abdominal cavity, these surgeries often require a steep head-down maneuver (HDM), which has a significant impact on the central and cerebral hemodynamics in addition to ventilation. Pneumoperitoneum and HDM increase the intracranial pressure [6–10], which may impair intracranial microcirculation. Cerebral deoxygenation during surgery is known to be associated with an increased risk for postoperative cognitive dysfunction [11]. Although studies using near-infrared spectroscopy have suggested preserved cerebral oxygenation in the brain during pneumoperitoneum and HDM [12, 13], the incidence of postoperative cognitive dysfunction remains high, ranging from 11.9 to 45.8% [14–16]. Furthermore, several case reports have indicated that robot-assisted surgery with pneumoperitoneum with HDM can lead to extensive brain edema [17, 18]. These adverse postoperative consequences may be attributable to impaired intracranial microcirculation caused by pneumoperitoneum and HDM. Therefore, it is important to understand and control intracranial microcirculation to secure cerebral perfusion during these surgeries and general anesthesia. However, no study has directly investigated the regulation of the cerebral microvasculature during pneumoperitoneum and HDM.

In the present study, we investigated the direct effects of pneumoperitoneum and steep HDM on cerebral microvasculature using the cranial window technique in rabbits. Furthermore, we also investigated the effects of pneumoperitoneum and HDM on the brain water content. We hypothesized that pneumoperitoneum with or without HDM dilates pial arterioles and evaluated whether these maneuvers cause brain edema.

## Methods

The experimental protocol was reviewed and approved by the University of Yamanashi Animal Care Committee (Protocol approval number: A2–18). The study was carried out and is reported in compliance with the ARRIVE guidelines.

### Animals

Male Japanese white rabbits (Kbs:JW) weighing 2.4–3.0 kg were purchased from Kitayama Labes (Nagano, Japan). The rabbits were housed at 23 ± 2 °C under a 12-h light–dark cycle with free access to standard food and water. All the experiments were performed between 9 a.m. and 5 p.m. under normal room light and temperature (23 ± 2 °C) conditions.

### Anesthesia and experimental preparation

After gaining intravenous access through an ear vein, the rabbits were anesthetized with pentobarbital sodium (20 mg/kg) and rocuronium bromide (3.3 mg/kg), followed by a continuous infusion of pentobarbital sodium (5 mg/kg/h) and rocuronium bromide (3.3 mg/kg/h). A tracheostomy was performed, and a 3.5-mm endotracheal tube was inserted. The animals were mechanically ventilated with 50% oxygen and 50% nitrogen. End-tidal carbon dioxide (EtCO_2_) levels were continuously monitored using a capnogram (Vamos; Drager Medical, Tokyo, Japan). An arterial catheter was inserted into the femoral artery to monitor arterial blood pressure and heart rate, and to measure blood gas parameters (partial pressure of carbon dioxide [PaCO_2_] and oxygen [PaO_2_], pH, base excess, Na^+^, K^+^, and glucose). Based on the EtCO2 monitoring, PaCO_2_ was attempted to be maintained between 35 and 45 mmHg. Rectal temperature was constantly monitored and maintained at 39.0 ± 1.0 °C using a heating blanket.

### Cranial window installation

The closed cranial window method was used to visualize the cerebral microvasculature as previously described [19, 20]. Briefly, after aseptic preparation with 70% alcohol, a longitudinal scalp incision was performed over the parietal bone. An 8-mm diameter hole was made in the parietal bone using a dental microdrill. The dura and arachnoid membranes were cauterized and excised to visualize the pial blood vessels. A glass ring was positioned over the hole and secured using bone wax and dental acrylic. The space under the window was filled with artificial cerebrospinal fluid (aCSF), and two polyethylene catheters were inserted. One of the catheters was connected to a reservoir bottle containing aCSF that was continuously bubbled with 5% CO2 in air. The aCSF was suffused at 0.1 mL/min. The other catheter served as an outlet for the aCSF. The outlet catheter exit was placed approximately 5–6 cm above the right atrium. The composition of the aCSF was as follows: Na^+^: 151 mEq/L; K^+^: 3.5 mEq/L; Ca^2+^: 2.5 mEq/L; Mg^2+^: 1.3 mEq/L; HCO_3_^−^: 25 mEq/L; urea: 40 mg/dL; and glucose: 65 mg/dL.

### Measurement of pial arteriolar diameter

The pial vasculature was visualized using a microscope (VH-5000; Keyence, Osaka, Japan) attached to a video capture unit (VH-E500, Keyence) and digital video analyzer (VH- H1A5, Keyence). The microscope was positioned and fixed over the cranial window to secure the same observational view and measure the same portion of the arterioles throughout the experiment. A series of still images of the pial vasculature was captured, and the pial arteriolar diameters were measured when the arterioles constricted (end-diastole to early systole). Two measurements from two separate arterioles (one measurement per arteriole at each time point) were recorded and averaged. Changes in the pial arteriolar diameters were reported as relative changes from the baseline value by defining the baseline as one.

### Pneumoperitoneum and HDM

Rabbits were randomly allocated to the control, pneumoperitoneum alone (P), and pneumoperitoneum with HDM (P + HDM) groups (*n* = 7 each). Following asepsis, a small abdominal incision was made in the umbilical region in all three groups, and a thin polyethylene catheter was inserted into the abdomen of rabbits in the P and P + HDM groups. Carbon dioxide was introduced via the catheter, and the insufflation pressure was maintained at 10 mmHg to achieve pneumoperitoneum for 120 min. In the P + HDM group, 30° HDM was initiated upon insufflation of pneumoperitoneum and until desufflation. Pial arteriolar diameter and hemodynamic parameters were measured at the following time points: before abdominal incision (baseline); immediately after insufflation (0 min); 20, 40, 60, 80, 100, and 120 min after insufflation; and 20 min after desufflation. Rabbits in the control group were evaluated for pial arteriolar diameter and hemodynamic parameters at the corresponding time points (baseline, 0, 20, 40, 60, 80, 100, 120, and 140 min after abdominal incision). Blood gas analysis was performed at these time points. All animals were euthanized by intravenous administration of 150 mg/kg pentobarbital sodium after the last measurement.

### Evaluation of brain water content

After euthanasia, the cranial window was removed from the parietal bone, and approximately 0.2 cm^3^ of the brain tissue from the parietal cortex was harvested. The wet weight of the brain tissue was measured using a digital scale, and the tissue was freeze-dried for 72 h to measure its dry weight [21]. The brain water content was calculated as follows: (wet weight - dry weight) / wet weight × 100%.

### Statistical analysis

Statistical analyses were performed using GraphPad Prism 9 (GraphPad Software, San Diego, CA, USA) and R version 4.0.4 (R Foundation for Statistical Computing, Vienna, Austria). One-sample Kolmogorov–Smirnov and Shapiro–Wilk normality tests were used to evaluate the normal distribution, and *p* > 0.05 was confirmed in both tests for all datasets. Two-way analysis of variance (ANOVA) was used to analyze pial arteriolar diameter and other variables. The intergroup comparison at each time point and intragroup comparisons (each time point vs. baseline) were conducted using Bonferroni’s multiple comparisons test. One-way ANOVA was used to analyze the brain water content. Power analysis indicated that a sample size of 7 animals per group was sufficient to achieve 80% power, with an α level of 0.05 to detect a mean difference of 15% in arteriolar diameter (G*Power version 3.1.9.3). As a post-hoc analysis, correlations between changes in pial arteriolar diameter and those in mean arterial pressure (MAP) were analyzed using Spearman’s rank correlation. For this analysis, data from P and P + HDM groups at the initiation (baseline–0 min) and termination (120–140 min) of pneumoperitoneum (and HDM) were used. All values are presented as mean ± standard deviation. A *p* < 0.05 was considered to be statistically significant. For the multiple comparison analyses, the *p* values were adjusted for multiplicity (adjusted *p* value, the smallest familywise significance level at which a particular comparison will be declared statistically significant as part of the multiple comparison testing).

## Results

**Fig. 1:**
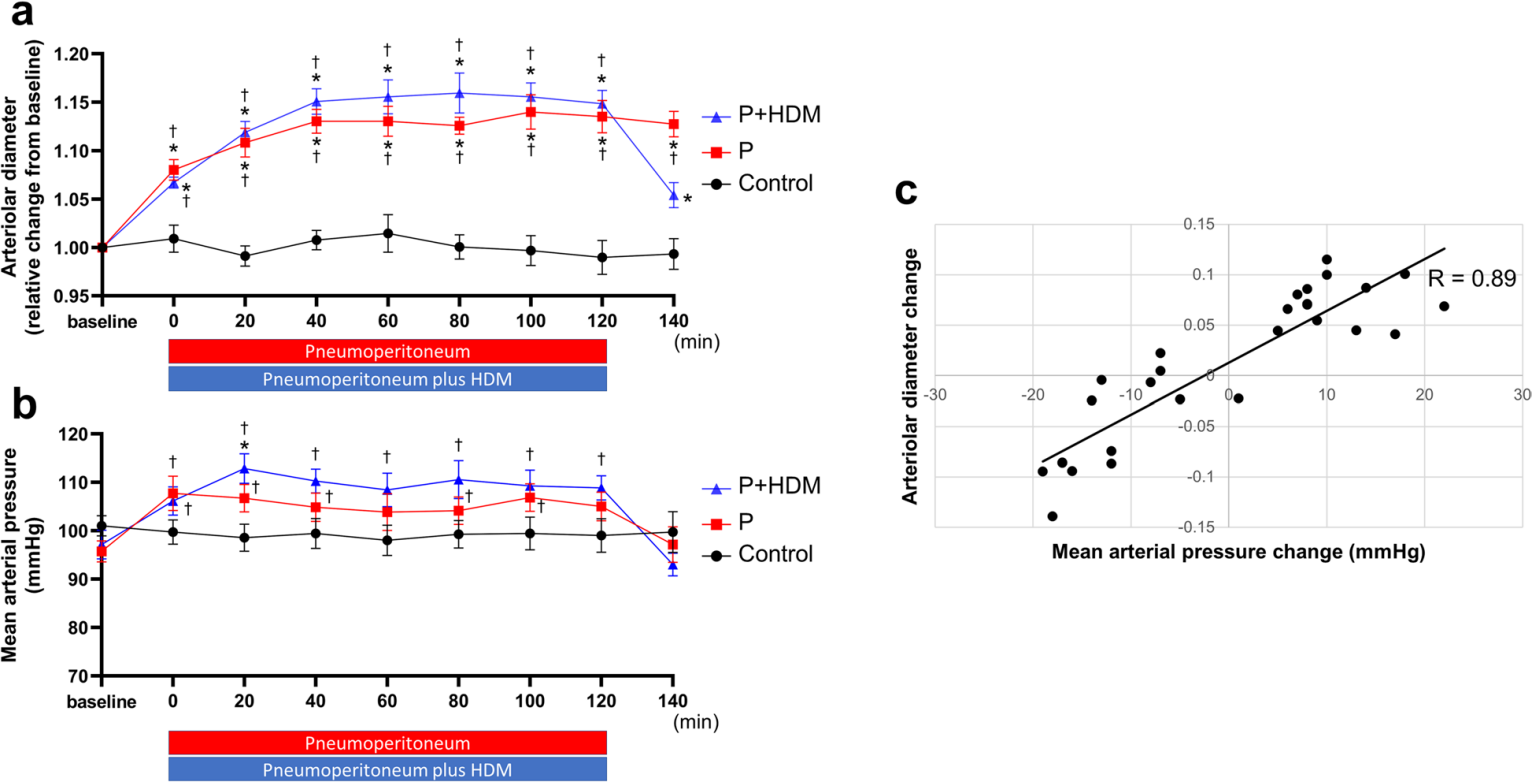
**a** Changes in pial arteriolar diameter. **b** Changes in mean arterial pressure. Values are mean ± standard deviation (*n* = 7 each). P + HDM: Pneumoperitoneum and head-down maneuver; P: Pneumoperitoneum only. *: *p* < 0.05 (vs. control); †: *p* < 0.05 (vs. baseline). **c** Scatterplot showing the correlation between the changes in mean arterial pressure and pial arteriolar diameter at initiation (baseline–0 min) and termination (120–140 min) of pneumoperitoneum (and HDM)

Pial arteriole diameter was increased at time points 0 to 120 min as compared to the baseline in the P and P + HDM groups (Fig. 1a). There was no significant difference in the arteriolar diameters between the P and P + HDM groups at any time point during pneumoperitoneum (0–120 min). After discontinuation of pneumoperitoneum and HDM, the diameter of the pial arterioles returned to the baseline level in the P + HDM group (1.05 ± 0.03, *p* = 0.0906, vs. baseline). In contrast, pial arterioles remained dilated as compared to the baseline level in the P group after discontinuation of pneumoperitoneum (1.13 ± 0.04, *p* = 0.941, vs baseline). The diameter of the pial arterioles did not change in the control group rabbits throughout the experiment.

As shown in Fig. 1b, rabbits in both P and P + HDM groups exhibited a significant increase in MAP during pneumoperitoneum (and HDM) compared to the control group. There were no differences in MAP between the P and P + HDM groups during pneumoperitoneum (and HDM). Twenty minutes after discontinuation of pneumoperitoneum (and HDM), MAP was similar to the baseline level in both P and P + HDM groups (P: 97 ± 10 mmHg, *p* > 0.999 and P + HDM: 93 ± 6 mmHg, *p* = 0.168, vs. baseline). Changes in arteriolar diameter correlated with the changes in MAP (correlation coefficient = 0.89, *p* < 0.001, Fig. 1c). The MAP did not change in the control group rabbits throughout the experiment.

PaCO_2_ was maintained at approximately 35–45 mmHg in all the groups throughout the experiment, and no intra- or intergroup differences were observed (Table 1). Heart rate, PaO_2_, Na^+^, K^+^, and glucose levels were not significantly different between the groups. P and P + HDM groups had lower pH and base excess than those in the control group.

**Table 1:** Hemodynamic parameters and blood gas data

	HR	pH	BE	PaCO_2_	PaO_2_	Na	K	Glucose
	(beats/min)			(mmHg)	(mmHg)	(mEq/L)	(mEq/L)	(mg/dL)
**Control group (*n*** **= 7)**								
Baseline	279 ± 30	7.45 ± 0.03	2.3 ± 2.4	38.5 ± 2.8	197 ± 25	142 ± 2	3.4 ± 0.7	125 ± 7
0 min	269 ± 31	7.44 ± 0.03	2.4 ± 2.1	37.8 ± 2.8	202 ± 26	142 ± 1	3.3 ± 0.7	125 ± 11
20 min	246 ± 45	7.44 ± 0.02	3.0 ± 2.1	40.3 ± 2.7	206 ± 28	143 ± 2	3.3 ± 0.6	123 ± 9
40 min	243 ± 41	7.44 ± 0.03	2.7 ± 1.6	39.3 ± 3.5	207 ± 23	143 ± 2	3.1 ± 0.1	122 ± 11
60 min	251 ± 47	7.40 ± 0.03	3.7 ± 2.2	41.0 ± 1.9	209 ± 27	142 ± 2	3.2 ± 0.4	124 ± 11
80 min	271 ± 31	7.46 ± 0.11	3.9 ± 2.2	41.6 ± 2.3	206 ± 25	142 ± 2	3.2 ± 0.2	125 ± 12
100 min	291 ± 23	7.45 ± 0.03	4.0 ± 2.0	39.3 ± 2.5	204 ± 31	142 ± 1	3.2 ± 0.4	125 ± 13
120 min	286 ± 23	7.45 ± 0.02	3.7 ± 1.9	39.3 ± 3.4	206 ± 24	142 ± 1	3.1 ± 0.2	124 ± 12
140 min	290 ± 17	7.44 ± 0.02	3.4 ± 2.1	39.8 ± 2.4	203 ± 26	142 ± 2	3.2 ± 0.2	125 ± 12
**Group P (*n*** **= 7)**								
Baseline	279 ± 36	7.36 ± 0.04*	−3.6 ± 2.1*	38.8 ± 2.8	201 ± 14	142 ± 1	3.1 ± 0.5	125 ± 15
0 min	271 ± 39	7.35 ± 0.05*	− 2.7 ± 2.1*	41.9 ± 3.2	216 ± 17	142 ± 2	3.2 ± 0.4	133 ± 19
20 min	283 ± 26	7.36 ± 0.04*	− 3.0 ± 1.8*	39.7 ± 2.8	206 ± 22	141 ± 2	3.3 ± 0.5	123 ± 17
40 min	281 ± 24	7.36 ± 0.04*	− 2.6 ± 3.0*	40.1 ± 4.8	197 ± 27	142 ± 2	3.1 ± 0.3	117 ± 13
60 min	279 ± 29	7.37 ± 0.04*	−1.7 ± 2.6*	40.9 ± 2.2	201 ± 10	141 ± 1	3.3 ± 0.2	116 ± 9
80 min	287 ± 21	7.37 ± 0.04	− 1.9 ± 2.2*	40.6 ± 1.8	205 ± 24	141 ± 2	3.4 ± 0.2	117 ± 8
100 min	287 ± 14	7.36 ± 0.05*	−2.0 ± 2.8*	42.0 ± 2.4	201 ± 26	141 ± 2	3.4 ± 0.2	118 ± 10
120 min	290 ± 18	7.37 ± 0.05*	−1.3 ± 2.9*	41.2 ± 2.3	208 ± 22	141 ± 2	3.3 ± 0.3	119 ± 9
140 min	285 ± 24	7.41 ± 0.02*	− 1.0 ± 2.1*	37.2 ± 1.9	193 ± 34	141 ± 2	3.3 ± 0.2	117 ± 8
**Group P + H (*n*** **= 7)**								
Baseline	274 ± 27	7.35 ± 0.06*	−2.9 ± 2.9*	40.2 ± 2.7	196 ± 17	142 ± 1	2.9 ± 0.3	129 ± 14
0 min	264 ± 39	7.37 ± 0.04*	−1.9 ± 3.1*	40.8 ± 3.0	193 ± 18	143 ± 2	2.9 ± 0.5	126 ± 17
20 min	293 ± 23	7.37 ± 0.05*	− 2.0 ± 2.8*	40.5 ± 2.7	187 ± 27	142 ± 2	3.0 ± 0.3	129 ± 14
40 min	290 ± 26	7.37 ± 0.07	− 0.9 ± 3.2	41.6 ± 2.0	198 ± 16	142 ± 1	3.1 ± 0.3	120 ± 12
60 min	293 ± 24	7.37 ± 0.04*	−1.3 ± 3.3*	42.0 ± 2.4	192 ± 20	142 ± 1	3.2 ± 0.3	121 ± 8
80 min	301 ± 27	7.38 ± 0.05	− 0.4 ± 3.2*	41.3 ± 3.7	181 ± 22	142 ± 2	3.3 ± 0.4	122 ± 10
100 min	313 ± 22	7.40 ± 0.04*	− 0.4 ± 3.3*	38.9 ± 3.4	191 ± 25	142 ± 1	3.4 ± 0.4	126 ± 11
120 min	306 ± 22	7.39 ± 0.06	− 0.3 ± 3.8*	40.8 ± 3.8	185 ± 25	142 ± 2	3.3 ± 0.3	122 ± 11
140 min	296 ± 29	7.42 ± 0.04	− 0.6 ± 2.9*	36.8 ± 1.4	206 ± 11	142 ± 2	3.3 ± 0.3	125 ± 9

Values are expressed as mean ± SD

*P* pneumoperitoneum, *P + HDM *pneumoperitoneum with head-down maneuver, *HR *heart rate, *BE *base excess

*p < 0.05 (vs. control)

The brain water content was not significantly different between the groups (control: 80.1% ± 1.0%, P: 79.4% ± 1.5%, and P + HDM: 80.4% ± 1.0%, *p* = 0.651).

## Discussion

In this study, we found that pneumoperitoneum with or without HDM immediately caused pial arteriolar dilation. According to Poiseuille’s law, blood flow is proportional to the fourth power of the radius of the blood vessel. Therefore, the cerebral vasodilation observed in the current study indicates increased cerebral blood flow (CBF) during pneumoperitoneum, consistent with a previous human study [22]. Whether HDM increases CBF remains controversial. While Lovell et al. reported that the CBF increased with an 18° HDM [23], others reported that steady-state tilt has no effect on CBF [24]. In the present study, the pial arteriolar diameters in the P and P + HDM groups were similar, indicating that adding HDM to pneumoperitoneum has no additional effect on CBF. Rabbits that underwent pneumoperitoneum and HDM exhibited a decrease in pial arteriolar diameter upon discontinuation of the maneuvers. This observation is in agreement with a previous finding that CBF is elevated during pneumoperitoneum and HDM and that CBF rapidly returns to baseline when the patient’s position returns to supine [23]. Studies by our and another group have independently indicated that cerebral oxygenation in patients undergoing surgery with a combination of pneumoperitoneum and HDM is generally elevated [12, 14, 25, 26], indicating increased blood volume in the cerebral arterial component. These findings are consistent with the present results that the cerebral arterioles dilated during pneumoperitoneum and HDM.

Of the various physiological and hemodynamic parameters, PaCO_2_ is a crucial factor that regulates CBF. Insufflation with CO_2_ generally increases PaCO_2_, that causes dilation of cerebral microvessels and increases CBF. However, in the present study, ventilator settings were adjusted such that PaCO_2_ was maintained at 35–45 mmHg, and no significant changes in PaCO_2_ were observed during the experiment in any group. Besides PaCO_2_, hyperdynamic cardiovascular response may increase CBF during pneumoperitoneum [27, 28]. The cardiovascular reactions require some time to subside after the termination of pneumoperitoneum. Indeed, we observed persistent pial arteriolar dilation in rabbits that received only pneumoperitoneum even after discontinuing pneumoperitoneum. In contrast, the direct influence of a specific body position on the circulatory system would rapidly disappear after discontinuation of the position [23]. In the present study, pial arteriolar diameter returned to the baseline level after the termination of pneumoperitoneum and HDM. A strong correlation between changes in MAP and those in pial arteriolar diameter was observed, indicating that MAP may be a factor in the regulation of cerebral microcirculation.

We did not find a significant increase in brain water content between groups, suggesting that 2 h of pneumoperitoneum and 30° HDM did not cause brain edema. This is in agreement with the observation in humans that the zero-flow pressure, the theoretical pressure at which cerebral circulation ceases, did not change during P + HDM [29]. However, Doi et al. reported that the brain water content increased after 8 h of a 45° HDM in rabbits [30]. Although no significant histological changes were observed in their study, mild to moderate brain edema can be difficult to detect, and the results do not necessarily verify the absence of brain edema [31]. In addition, several case reports have described brain edema or neurological dysfunction following prolonged laparoscopic surgery with HDM [17, 18, 32].

This study has some limitations. It was conducted in rabbits, in which the relative vertical distance between the head and heart usually varies much less than in humans. Therefore, rabbits may have fewer protective mechanisms than humans to compensate for hydrostatic pressure alterations. In addition, the hydrostatic pressure changes during HDM are much smaller in rabbits than in humans. Considering these factors, our findings may not be directly applicable to humans. We also used relatively young male animals. Future studies should use animals of both sexes and of different ages to confirm the findings of the present study. Furthermore, although the dilated pial arterioles suggest an increase in CBF, since the blood velocity or cerebral oxygenation was not measured, the effect of pneumoperitoneum with or without HDM on CBF was not established in this study. Future studies should evaluate these parameters. In addition, we used pentobarbital for general anesthesia, which reduces CBF and cerebral metabolic rate of oxygen [33], and different results might have been obtained using different anesthetic agents. There were differences in arterial pH and base excess between groups at baseline, which could also have affected the results. Moreover, although PaCO_2_ was maintained at similar levels throughout the experiments, a trend towards higher PaCO_2_ could have been possible during pneumoperitoneum compared with the baseline in the P group rabbits. The subtle changes in PaCO_2_ could have affected the results. Regarding the statistical analysis, although we found a strong correlation between the changes in MAP and those in pial arteriolar diameter, there could have been confounding factors that were not measured that potentially influenced both of these parameters.

## Conclusions

The present study suggests that pial arterioles dilate immediately after pneumoperitoneum with or without HDM. The pial arterioles remain dilated after discontinuation of pneumoperitoneum alone, but constrict upon discontinuation of pneumoperitoneum plus HDM. Changes in arteriolar diameter correlated with changes in MAP. Pneumoperitoneum and HDM for 2 h did not cause brain edema in rabbits.

## Data Availability

The datasets used and/or analyzed during the current study are available from the corresponding author on reasonable request.

## Abbreviations

ANOVA: Analysis of variance
aCSF: Artificial cerebrospinal fluid
CBF: Cerebral blood flow
EtCO_2_: End-tidal carbon dioxide
HDM: Head-down maneuver
PaCO_2_: Partial pressure of carbon dioxide
PaO_2_: Partial pressure of oxygen
MAP: Mean arterial pressure
